# Uncovering the Reasons Behind COVID-19 Vaccine Hesitancy in Serbia: Sentiment-Based Topic Modeling

**DOI:** 10.2196/42261

**Published:** 2022-11-17

**Authors:** Adela Ljajić, Nikola Prodanović, Darija Medvecki, Bojana Bašaragin, Jelena Mitrović

**Affiliations:** 1 The Institute for Artificial Intelligence Research and Development of Serbia Novi Sad Serbia; 2 Faculty of Computer Science and Mathematics, University of Passau Passau Germany

**Keywords:** topic modeling, sentiment analysis, LDA, NMF, BERT, vaccine hesitancy, COVID-19, Twitter, Serbian language processing, vaccine, public health, NLP, vaccination, Serbia

## Abstract

**Background:**

Since the first COVID-19 vaccine appeared, there has been a growing tendency to automatically determine public attitudes toward it. In particular, it was important to find the reasons for vaccine hesitancy, since it was directly correlated with pandemic protraction. Natural language processing (NLP) and public health researchers have turned to social media (eg, Twitter, Reddit, and Facebook) for user-created content from which they can gauge public opinion on vaccination. To automatically process such content, they use a number of NLP techniques, most notably topic modeling. Topic modeling enables the automatic uncovering and grouping of hidden topics in the text. When applied to content that expresses a negative sentiment toward vaccination, it can give direct insight into the reasons for vaccine hesitancy.

**Objective:**

This study applies NLP methods to classify vaccination-related tweets by sentiment polarity and uncover the reasons for vaccine hesitancy among the negative tweets in the Serbian language.

**Methods:**

To study the attitudes and beliefs behind vaccine hesitancy, we collected 2 batches of tweets that mention some aspects of COVID-19 vaccination. The first batch of 8817 tweets was manually annotated as either relevant or irrelevant regarding the COVID-19 vaccination sentiment, and then the relevant tweets were annotated as positive, negative, or neutral. We used the annotated tweets to train a sequential bidirectional encoder representations from transformers (BERT)-based classifier for 2 tweet classification tasks to augment this initial data set. The first classifier distinguished between relevant and irrelevant tweets. The second classifier used the relevant tweets and classified them as negative, positive, or neutral. This sequential classifier was used to annotate the second batch of tweets. The combined data sets resulted in 3286 tweets with a negative sentiment: 1770 (53.9%) from the manually annotated data set and 1516 (46.1%) as a result of automatic classification. Topic modeling methods (latent Dirichlet allocation [LDA] and nonnegative matrix factorization [NMF]) were applied using the 3286 preprocessed tweets to detect the reasons for vaccine hesitancy.

**Results:**

The relevance classifier achieved an *F*-score of 0.91 and 0.96 for relevant and irrelevant tweets, respectively. The sentiment polarity classifier achieved an *F*-score of 0.87, 0.85, and 0.85 for negative, neutral, and positive sentiments, respectively. By summarizing the topics obtained in both models, we extracted 5 main groups of reasons for vaccine hesitancy: concern over vaccine side effects, concern over vaccine effectiveness, concern over insufficiently tested vaccines, mistrust of authorities, and conspiracy theories.

**Conclusions:**

This paper presents a combination of NLP methods applied to find the reasons for vaccine hesitancy in Serbia. Given these reasons, it is now possible to better understand the concerns of people regarding the vaccination process.

## Introduction

### Background

The COVID-19 pandemic has significantly disrupted the daily lives of individuals and the way in which organizations operate worldwide. One of the most effective strategies to tackle the COVID-19 pandemic is to achieve collective immunity through mass vaccination [1,2]. However, people have shown significant resistance and hesitancy to the global immunization process [3]. The World Health Organization (WHO) identified vaccine hesitancy as 1 of the top 10 threats to global health care in 2019 [4]. Therefore, the study of the public attitude toward the vaccination process is of utmost importance. In particular, it is useful to identify the prevailing beliefs and attitudes that may lead to a negative sentiment toward vaccination. According to WHO, many events have the potential to erode confidence in vaccines. Some of them are related to vaccine safety and adverse events following immunization, but some are related to social media stories or rumors [5], making it significant to analyze the beliefs, attitudes, and fears reflected in the user-generated content on social media.

This kind of research needs to be conducted regionally worldwide since attitudes of people from different world regions vary significantly [3,6]. This presents a fair challenge as numerous languages of small populations worldwide, Serbian being among them, lack electronic resources. Due to the rapid advancement of artificial intelligence and machine natural language processing (NLP), we believe it is now possible to tackle this challenge, and thus demonstrate a possible solution for the case of Serbian as an example. The main contribution of our work is in the application of a combination of NLP methods to a low-resourced language to discover hidden topics related to vaccine hesitancy with minimum data annotation.

The research community predominantly used Twitter to collect data on COVID-19 vaccination [7-22]. We also opted for this social media since this is the platform where users produce large amounts of data that can be used for analysis of perceptions and narratives [23], collective experiences, behaviors, and attitudes related to particular social events [24]. Additionally, Twitter provides an application programming interface (API) that enables easier extraction of data compared to other platforms [25]. The use of this API allowed us to collect 14,452 tweets related to vaccination in the Republic of Serbia. The collected data span from January 2021, right after the first COVID-19 vaccines got released, to June 2022. The goal of our research is to look for topics in the tweets that express negative attitudes toward vaccination, which we believe would be most revealing with regard to the reasons for vaccine hesitancy.

A part of the data set was manually annotated using 4 class labels: irrelevant, positive, negative, and neutral. This data set was used to train a sequential bidirectional encoder representations from transformers (BERT)-based classifier, which then served to automatically annotate the rest of the data. After gathering the set of tweets with a relevant and clear negative sentiment toward vaccination through both manual and automatic annotation, we conducted topic analysis in order to pinpoint the main reasons for vaccine hesitancy.

The aim of this study is to detect the main topics within tweets in Serbian that express a negative sentiment regarding COVID-19 vaccination under an assumption that these topics point to the main reasons for vaccine hesitancy in Serbia. This information can help local domain experts influence the public in a more informed way with regard to vaccination. Knowing why people, especially young people, are hesitant equips the key decision makers with the right tools for planning vaccination-oriented campaigns.

### Related Work: Tweet Classification

The length and impact of the COVID-19 pandemic led to a surge in user-generated pandemic-related content on Twitter. The ability to automatically classify that content using machine learning and deep learning methods became especially important when information about COVID-19 vaccines started appearing. Previous work on sentiment analysis and human papillomavirus vaccination [26-28], and vaccination in general [11,12], served as a base for research into automatic classification of the sentiments of COVID-19–related tweets.

In recent years, there has been a significant shift in the design of machine learning architecture for the purpose of short text classification. With regard to public opinions about vaccination, the most traditionally exploited idea is that of static text embeddings combined with classical machine learning methods [11,12]. Relatively recently, systems based on recurrent neural networks (RNNs) started being used for such purposes [27,28]. A new family of methods based on attention neural networks was introduced in 2017. Their self-attention mechanism efficiently captures long-range dependencies through the pretraining process by maximally using parallel computation algorithms and hardware [29]. This gives this method a significant advantage over its predecessors based on RNNs to produce context and morphosyntactic aware embeddings. Historically, the sequence-to-sequence transduction model was the original model with the attention mechanism [29], but soon after, the first encoder-only architecture capable of providing only embeddings was published under the acronym BERT [30].

With the rise in computational power, many researchers were able to apply BERT to COVID-19 and vaccination content in English and test its results against older methods, such as bidirectional long-short term memory, support vector machines, and naïve Bayes. BERT-based architecture proved to be superior both for binary sentiment, relevance, or misinformation classification [9,13,19,28] and for tertiary stance or sentiment classification [14,17,19], which prompted us to choose such architecture for our research.

The pretraining strategy for BERT is usually defined as a masked language modeling task, which resembles the autoencoders, and a next sentence prediction task [30]. The most recent proposal for a pretraining strategy is the Efficiently Learning an Encoder that Classifies Token Replacements Accurately (ELECTRA) approach, where the BERT model is trained as a discriminator rather than a generator. This method was used to train BERTić [31], the first BERT-based model for South Slavic languages and the model we used to develop our classifiers.

BERTić has already been tested on tasks of short text classification for Serbian. Batanović [32] compared the results of BERT and BERTić to several linear classifiers on different classification tasks for movie reviews and showed that BERTić was the most optimal model for the tasks of binary and 4-class polarity classification. Mochtak et al. [33] worked on the tasks of ternary (negative-positive-neutral) and binary (negative and other) classification of sentences from parliamentary proceedings for Croatian, Serbian, and Bosnian. They tested several models: fastText with pretrained CLARIN.SI word embeddings, Cross Lingual Model – Roberta (XLM-Roberta), cseBERT, and BERTić. The best results were obtained with BERTić for all 3 languages. To the best of our knowledge, our work is the first attempt to apply BERTić to the classification of tweets in Serbian.

### Related Work: Tweet Topic Modeling

Since the beginning of the COVID-19 pandemic, researchers have attempted to use topic modeling to determine public attitudes toward various aspects of the pandemic [7,10,34], particularly vaccination [8,15,16,20-22,34-36]. Topic modeling is a method that allows grouping of documents into a predetermined number of topics. As a method that does not require any supervision or prior data labeling, it is popular for detecting hidden attitudes in a large variety of documents. Historically devised for longer texts, topic modeling has been confronted in recent years with the challenge of unveiling topics in short, unstructured, and informal social media comments [37]. Despite proposing methods to specifically tackle short text [38,39], and aggregating shorter texts into pseudodocuments before applying topic modeling [40-42], classical topic modeling methods, such as latent Dirichlet allocation (LDA) [43] and nonnegative matrix factorization (NMF) [44], remain the preferred methods when tackling tweets and social media comments in general.

LDA is a generative probabilistic model for collection of discrete data and is therefore used for discovering latent semantic structures from text corpora by capturing the pattern of co-occurrence of words at the document level. It has been especially widely used during the COVID-19 pandemic to determine the most discussed topics [7,10], correlate the vaccination stance and events in the media [8,17] or other spatiotemporal factors [16,36] and determine vaccine hesitancy topics [21,35], the general sentiment toward COVID-19 vaccines [20], and its changes over time [15].

NMF is a nonprobabilistic method based on matrix decomposition actively used for topic modeling [44,45]. It has also been applied to the theme of COVID-19 to determine the main pandemic health effects [34] and the public sentiment toward vaccination [22]. Compared to LDA, which gives more general descriptions of broader topics [46], the architecture of NMF enables it to find more detailed, clear-cut, and coherent topics [37,46,47]. Chen et al [18] even claim that NMF can learn from data similarly to the way humans do, which makes its results more easily interpretable than in the case of LDA.

Given that the 2 models approach the data and the topics differently, we decided to use a combination of their results in order to determine the final list of topics in our research.

Even though substantial work has been conducted on sentiment analysis for Serbian [48-52], to the best of our knowledge, this is the first attempt to apply topic modeling to Serbian.

## Methods

### Study Design

To study the attitudes and beliefs behind vaccine hesitancy, we first collected 2 batches of tweets that mention some aspect of COVID-19 vaccination. We manually annotated the first set of tweets as either relevant or irrelevant with regard to the COVID-19 vaccination sentiment and then annotated the relevant ones as positive, negative, or neutral. In addition, we manually searched for topics related to vaccine hesitancy in the negative tweets.

To augment this initial data set, we used the annotated tweets to train a sequential BERT-based classifier for 2 tweet classification tasks. In the first task, the classifier distinguished between relevant and irrelevant tweets. In the second task, the classifier took the relevant tweets as input and classified them as negative, positive, or neutral. We used this sequential classifier to annotate the second batch of tweets. We then combined the 2 data sets and applied 2 topic modeling methods (LDA and NMF) to them in order to detect the reasons for vaccine hesitancy.

**Figure 1:**
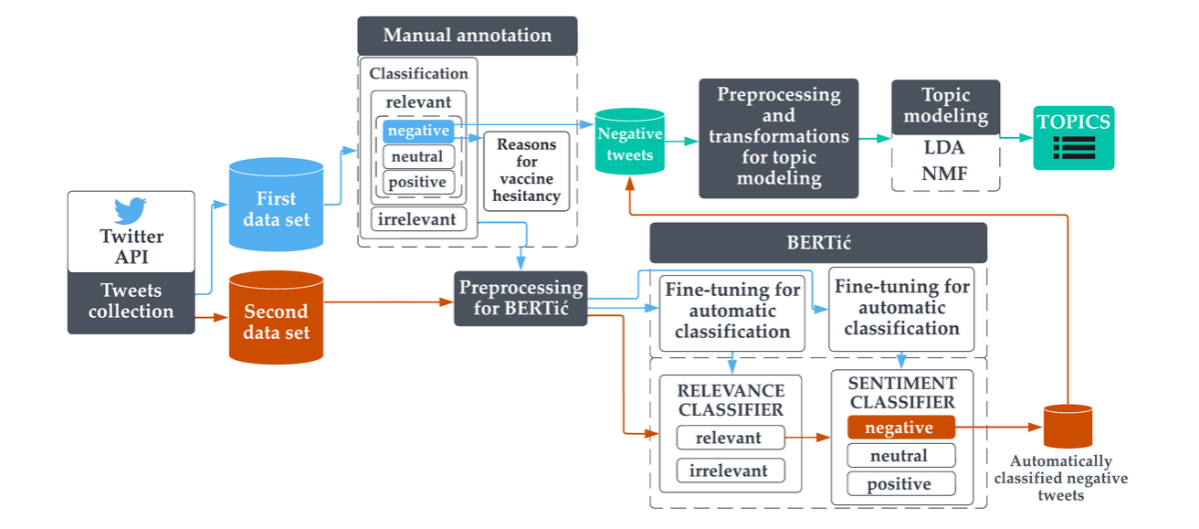
Tweet classification and topic modeling pipeline. API: application programming interface; BERT: bidirectional encoder representations from transformers; LDA: latent Dirichlet allocation; NMF: nonnegative matrix factorization.

This entire pipeline is presented in Figure 1. Each of the individual steps is described in detail in the following subsections.

### Data Collection and Annotation

We used the *Twarc* Python library [53] to extract the data in Serbian (in Cyrillic and Latin scripts) from the Twitter streaming API. The collection of tweets was divided into 2 phases, resulting in 2 subsets of data.

The first data set consisted of 8817 tweets collected between January 1 and November 23, 2021. Since the purpose of this data set was to reflect the opinions and topics of the citizens of Serbia, the query included the condition that the tweets either contain the location of the Republic of Serbia or be written in Serbian. We first tested the search using relevant hashtags (#COVID-19, #vakcina, etc), which did not yield enough tweets, because hashtags with Serbian words on this topic are not frequently used. For that reason, we based our search on keywords relevant to the topic of vaccination. The query consisted of all the writing and morphological variations for COVID-19 mutually connected with an OR operator (eg, “COVID-19” OR “corona” OR “kovid”) and all the writing and morphological variations for the words “vaccine” and “vaccination,” including vaccine types (“vakcina” OR “moderna” OR “fajzer”) in Latin and Cyrillic scripts. This enabled a search of all the tweets that were related to both COVID-19 and vaccines. Retweets were excluded from the search.

This entire data set needed to be annotated in order to train the classifiers. We compiled a detailed set of rules according to which the annotators conducted the labeling. The labels for the positive and negative sentiments were assigned to tweets with the respective type of attitude toward vaccination. A neutral sentiment was used for neutral attitudes about the topic but also for tweets that did not convey an explicit attitude of the user but contained some information about the topic. This included facts about COVID-19 vaccination, available doses or vaccination dates, objective questions about vaccination backed by the user’s obvious intention to seek other people’s opinion and information, jokes without attitude, and posting of neutral media headlines without additional personal comments. Furthermore, the annotators used a special class for irrelevant tweets, such as those containing an unclear or vague attitude. This class also included tweets that consisted of an external link and some user comments related to the content of the link, which was not sufficient to capture their attitude toward vaccination, because the links were not the subject of this analysis. The subjects of the annotation were text content and hashtags.

For the first 500 (5.7%) tweets, all the authors of this paper conducted the labeling and amended the initial set of rules through mutual discussion on the confused examples. The rest of the data set was individually and separately annotated by 2 annotators using the defined guidelines. After the whole data set was labeled, the Cohen *κ* score was 0.57 for all 4 classes, 0.67 for the 3 sentiment classes, and 0.73 for the positive and negative classes. The main point of disagreement between the annotators was in assigning the “neutral” versus the other 2 sentiment labels and the “irrelevant” versus the “relevant” label (positive, negative, and neutral), which was resolved by an author of this paper who was most involved in the COVID-19 vaccine discussion. The result was a data set of 5791 (65.7%) relevant tweets (irrelevant tweets=3026, 34.3%), divided into 3 sentiment classes. The statistics of the first subset can be seen in Table 1.

In addition to defining the sentiments of relevant tweets, the annotators separately indicated the topics that were prevalent in the negative tweets. The number of these topics was later used to set the upper limit for testing the optimal number of topics for the topic modeling methods.

The second subset of data was collected for the period from November 23, 2021, to June 6, 2022. After the first phase of tweet collection, we concluded that filtering the tweets by specifying the location and the Serbian language severely limited the number of tweets available for collection, so we decided to take a different approach.

**Table 1:** Vaccine hesitancy data set statistics for the relevant tweets in batch 1 (N=5791).

Sentiment class	Tweets, n (%)
Negative	1770 (30.6)
Positive	1965 (33.9)
Neutral	2056 (35.5)

Since the search condition regarding location can only be satisfied if the user shares the location at the time the tweet is published, which does not often seem to be the case for people from Serbia, this operator significantly limits the collection of tweets and excludes many potential results. Several problems occur when using the language operator. When Serbian is specified as the language, Cyrillic is the default script, so the collection of tweets written in Latin is omitted, as noticed in Ref. [49]. In fact, the Twitter API sorts out most of these tweets as an undefined language. In addition, some of the tweets collected in Cyrillic are in Northern Macedonian instead of Serbian. Therefore, we decided to exclude these 2 operators this time. As a result, our initial data set contained tweets in languages close to Serbian (Russian, Czech, Northern Macedonian, etc), which we filtered out using the language recognition library for Python *langID* [54].

This clean data subset consisted of 5635 tweets in Serbian. As this subset was meant to be used to test the performance of our classification model, it was not labeled by human annotators. The total number of tweets in both batches was 14,452.

### Automatic Tweet Classification

Deciding which tweet contains a negative sentiment is not a straightforward task. In our data set described in the previous subsection, about two-thirds of the total number of gathered tweets have an attitude toward vaccination, and only a subset of these tweets has a negative sentiment. We assumed that our data set was representative enough and therefore concluded that any further pipeline must contain automatic filtration of tweets into negative-sentiment tweets with sufficient relevancy in order to be able to automatically detect a large number of negative tweets for further analysis. With this in mind, we decided to develop a deep learning classifier that could detect relevant tweets with a sufficiently clear negative attitude toward the vaccination process. To build both classifiers, we used BERTić, a BERT-based model for South Slavic languages [31]. Instead of pretraining BERT from scratch on a much larger corpus of tweets [55], we used the annotated data to fine-tune and test BERTić on a downstream task of short text classification.

The classifier consists of 2 sequential parts. The first part filters tweets based on their relevance to the topic, and the second part filters tweets based on their sentiment. The second classifier takes as input the tweets that have passed the first filter for relevancy. We considered unifying these 2 classifiers into a single BERT architecture with an increased number of classes but abandoned this idea due to prominent class imbalance. The most interesting discussion arose for the boundary between irrelevant tweets and neutral-sentiment tweets. This boundary had to be introduced clearly through the annotation process. It was intuitively clear that class separation efficiency between the neutral class and the positive and negative classes would be sharper if we forced training only on the tweets that indeed had vaccines as the main topic but had no clear sentiment. This was our main reasoning behind the serialization of the classifiers.

The minimum preprocessing steps that we took before the training consisted of switching to the Latin script for all the tweets (using the *srtools* Python library [56]); restoring the diacritics (using the *classla* Python library [57]); removing the mentions, links, emojis, and noninformative hashtags; and transforming the remaining hashtags into words using regular expressions. We trained our algorithm on only 1 iteration of the annotation process because we also wanted to analyze possible human annotation errors and the robustness of the algorithm to the quality of annotation.

For the relevance classifier, the annotated data set was split into training, validation, and test sets according to the 80%:10%:10% ratio. The total number of examples in this data set was 8817. The validation set was used to choose the most optimal network solution among the maximum number of 6 training epochs.

For the sentiment polarity classifier, we developed a set of 5791 relevant tweets, which we split according to the 80%:10%:10% training:validation:test ratio.

The number of epochs and batch size were chosen to be optimal for a fixed validation set, which may result in a slight but acceptable bias. This is justified by the recommended values of these hyperparameters given in the original paper describing the BERT model [30], namely 4 epochs and a training batch size of 16 tweets.

### Topic Modeling

To uncover the reasons for vaccine hesitancy, we used 2 topic modeling methods on the data set of negative tweets: LDA and NMF. We decided to use these 2 models to compare the topics generated by completely different approaches.

For LDA, we used the implementation of Hoffman et al [58] and an open source *Gensim* Python library [59]. For NMF, we used the *sklearn* NMF decomposition the way it was implemented by Cichocki and Phan [60].

Before applying the topic modeling methods, we needed to go through several preprocessing steps to remove noise and reduce the space for topic modeling. The preprocessing pipeline consisted of switching from Cyrillic to Latin script; removal of URLs, mentions, numbers, new lines, emojis, images, special characters, etc; tokenization; lemmatization; and removal of stop words. We converted the tweets to Latin script using the *srtools* Python library, while tokenization and lemmatization were conducted using the *classla* pipeline for nonstandard Serbian. We removed the URLs, mentions, etc, using regular expressions. We used the list of stop words described by Marovac et al [61], which we extended with all the alternative names for COVID-19 and derivatives of the word “vaccine.” These terms naturally appear in most tweets since we applied them as our Twitter search keywords.

#### Building the Models

Both LDA and NMF require certain data set transformations. The transformations required to create the LDA model first include the creation of a vocabulary in the form of a list of unique words represented as integers. The next step is the pruning process: removing low- and high-frequency words. The final step is creating a corpus of all tweets as bag-of-words features. After these initial steps, we applied filters that excluded all the words that appeared in less than 3 tweets and more than 85% of tweets and limited the dictionary to 1000 terms. We chose to limit the dictionary since using more than 1000 terms resulted in less coherent topics. Additionally, a large dictionary allowed for less significant words to become more significant inside topic keywords due to the inability to quantify the importance of words.

For the NMF model, we used the term frequency–inverse document frequency (TF-IDF) transformation of the normalized text and applied the same filters as for LDA: we excluded words that appeared in less than 3 tweets and more than 85% of tweets and limited the dictionary to 1000 terms. We experimented with using several different combinations of filters for both models, which did not lead to significant changes in topics for the NMF model, but it did in the case of LDA. In general, NMF showed greater topic stability with the change in the dictionary size.

Each of the topic modeling methods requires a predefined number of topics. We calculated that number by tuning the model parameters and choosing the number of topics and parameters that yielded the highest coherence score value (c_v). The c_v score ranges from 0 to 1 and measures the co-occurrence of words in a topic inside the corpus. We opted for c_v as a metric since it increases monotonously with an increase in the number of topics, unlike another customarily used topic similarity metric, u_mass, which reaches the peak for a smaller number of topics and then decreases with an increase in the number of topics. When testing the models for the number of topics, we set the parameter *α* to “auto,” which made the model learn an asymmetric prior from the corpus.

In addition to c_v, we used another similarity metric, namely the Jaccard similarity coefficient. The Jaccard similarity coefficient ranges from 0 to 1 and measures the topic overlap. The lower the Jaccard similarity coefficient and the higher the c_v value, the more optimal the number of topics. Since c_v increases with an increase in the number of topics, which was not proven adequate for our data set, we applied the Jaccard similarity coefficient to normalize the number of topics. We set the limit for the optimal number of topics for both models to 15, as that was also the number of topics initially identified by human annotators.

**Figure 2:**
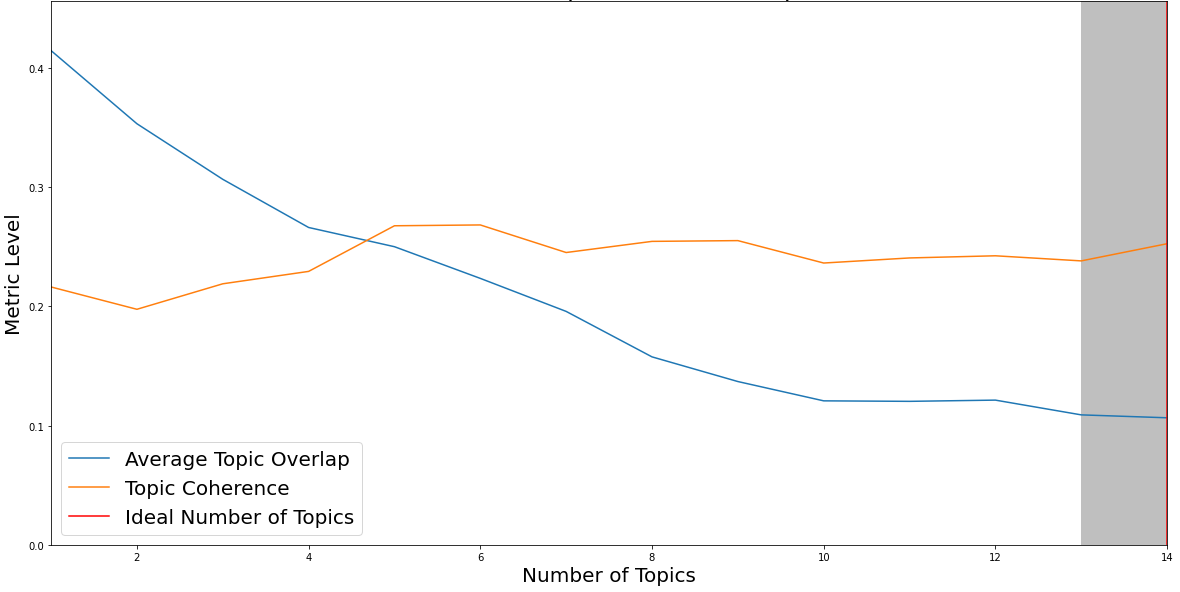
Optimal number of topics according to the coherence score value (c_v) and the Jaccard similarity coefficient for LDA. LDA: latent Dirichlet allocation.

After applying both c_v and Jaccard similarity coefficient metrics, the resulting optimal number of topics for LDA proved to be 14 (see Figure 2).

**Figure 3:**
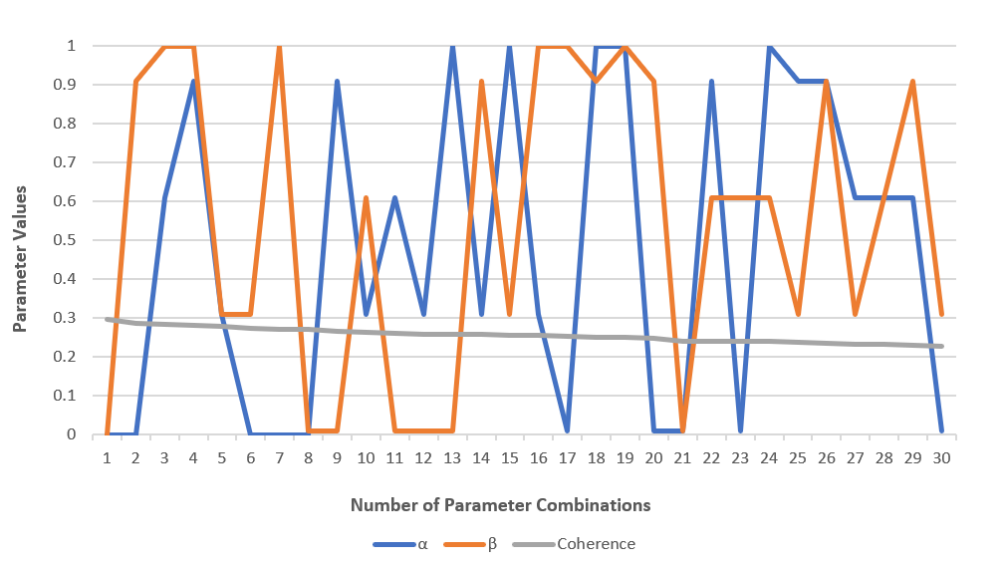
The c_v score for different values of *α* and *β* for 14 LDA topics. The “asymmetric” value is represented as 0 and the “symmetric” value as 1. LDA: latent Dirichlet allocation.

To obtain cluster assignments, LDA uses 2 probability values: *P*(word|topics) and *P*(topics|documents). In the *Gensim* model, parameters *α* and *β* affect these 2 probabilities. The *α* parameter is an a priori belief on document-topic distribution, while *β* is an a priori belief on topic-word distribution. After determining the optimal number of topics, we tuned these 2 parameters to obtain the best distribution of keywords per topic (see Figure 3). We made the model for the first 5 best-ranking combinations of *α* and *β*, and by manually comparing the topics, we chose the second one as best, which was *α*=“asymmetric” and *β*=0.91. A high value of *β* means that the topic can be assigned to more words. This was justified, given the nature of the data set focused on a narrow field where the same words often appear in different contexts, which makes the topics more similar based on the words they contain.

**Figure 4:**
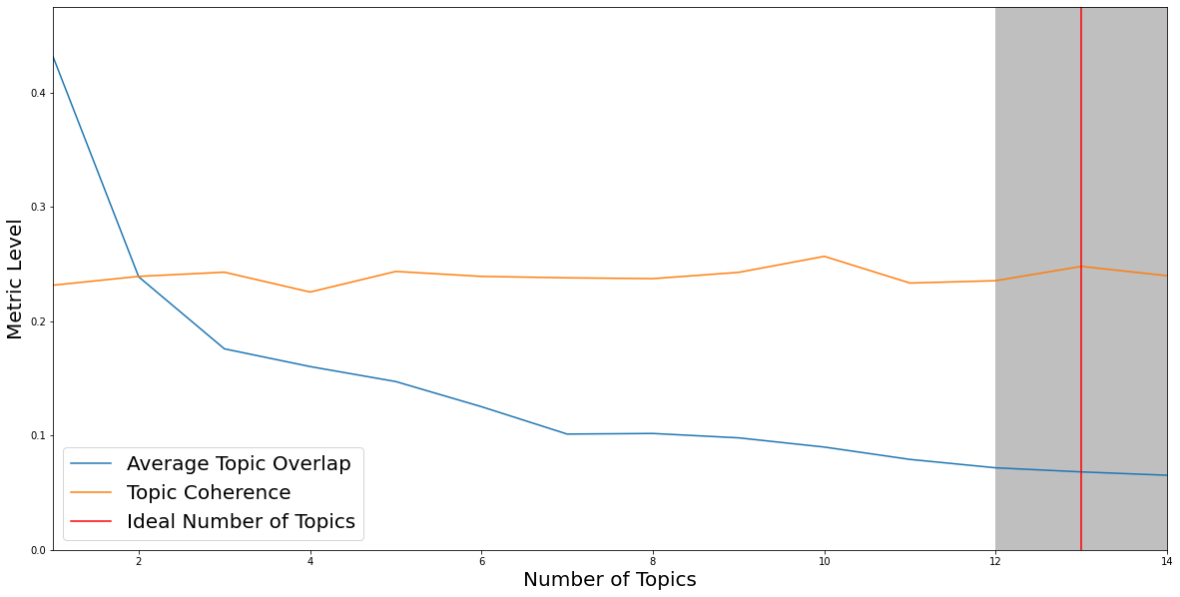
Optimal number of topics according to c_v and the Jaccard similarity coefficient for NMF. NMF: nonnegative matrix factorization.

After applying c_v and Jaccard similarity coefficient metrics, the resulting optimal number of topics for NMF proved to be 13 (see Figure 4).

**Figure 5:**
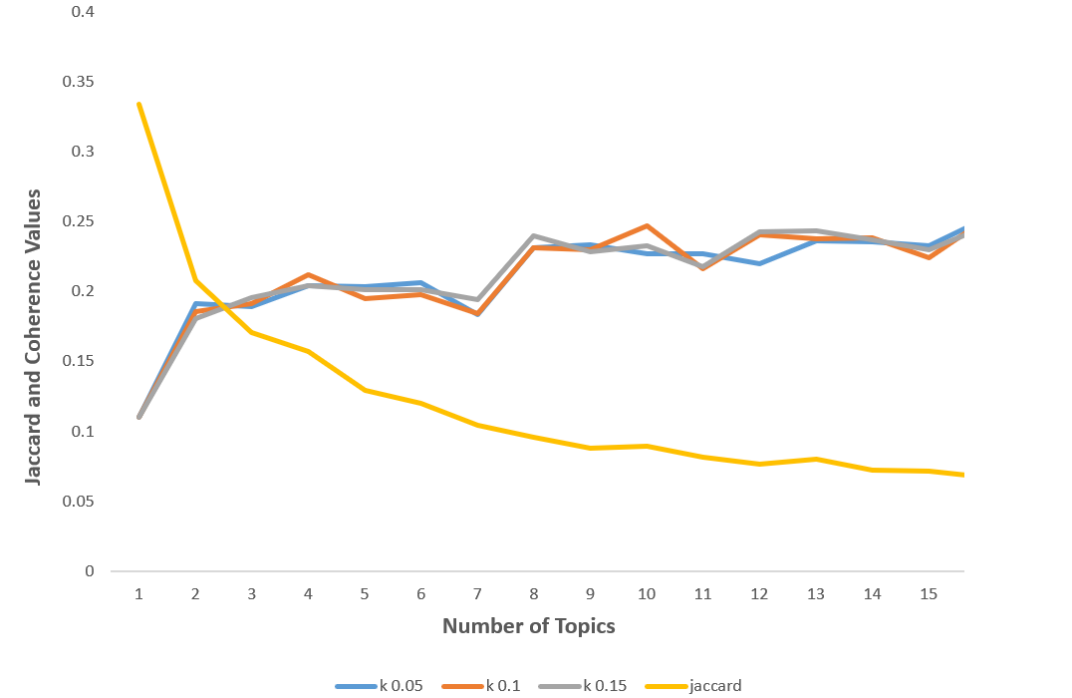
The c_v score and the Jaccard similarity coefficient for different learning rates (NMF). NMF: nonnegative matrix factorization.

For the NMF model, we used an input document-term matrix normalized with TF-IDF. The matrices into which the starting document-term matrix is decomposed are document-topic and topic-term matrices. We obtained the starting values of these 2 matrices by using singular value decomposition initialization presented in Belford et al [62], which is suitable for sparse data. For the fast convergence rate, we used coordinate descent solver-cd in *sklearn*. We tested the *κ* parameter, which determines the model convergence speed, and concluded it did not significantly affect coherence (see Figure 5). We chose a *κ* learning rate of 0.1, limited the number of iterations to 500, and set the random state to 42. We used the default value of 1e-4 for the tolerance of the stopping condition, and we did not use regularization parameters.

## Results

We grouped the individual results of automatic classification and topic modeling into 2 separate subsections, a*utomatic tweet classifier* and *topic modeling*.

### Automatic Tweet Classifier

We designed a sequential tweet classifier consisting of 2 BERTić classifiers. The first classifier was binary, and it decided whether a tweet was relevant for further analysis, while the second classifier performed the task of ternary classification and decided the type of sentiment associated with the tweet.

#### Relevance Classifier

The relevance classifier detected whether a tweet was relevant enough to be considered as an opinion about vaccines. Usually, irrelevant tweets are strongly related to epidemics and politics but without a clear attitude toward vaccination. We found that the political attitudes of Twitter users often mask attitudes toward vaccination. We decided to label extremely complex examples with completely masked attitudes as irrelevant, because it was obvious that users were frustrated by some other issues rather than by vaccination itself.

The algorithm was tested on 10% of the total number of tweets, which in this case was 882 tweets. The outright accuracy was 94.7%. The irrelevant class was imbalanced according to the 35%:65% ratio. However, after test set reannotation, many of the tweets were labeled as relevant, which shifted this imbalance below 30% for the irrelevant class; thus, we obtained lower *F*- and recall scores for the irrelevant class, valued at 0.91 and 0.86, respectively. The *F*-score for the relevant class was above 0.96. All the scores can be seen in Table 2.

The biggest issue was to come to a conclusion about the exact semantic boundary between the irrelevant tweets and the relevant tweets with a neutral sentiment. A neutral sentiment may also be understood as no sentiment, and thus irrelevant.

**Table 2:** Confusion matrix and *F*-scores for the relevance classifier.

Class	Irrelevant (predicted)	Relevant (predicted)
Irrelevant	225	35
Relevant	12	610
*F*-score	0.91	0.96

#### Sentiment Polarity Classifier

The sentiment polarity classifier took as input only relevant tweets and output their sentiment toward vaccination.

The accuracy of the model on the test set was about 85.7% (see Table 3).

Most of the confused examples fell between the neutral and the other 2 classes. Recall was the lowest for the positive class, with a value of 0.82. By careful inspection, we found no systematic error tendency for the algorithm or the annotators to confuse the positive class. Thus, the lower recall for the positive class is a consequence of a slightly imbalanced data set against the number of positive examples, as can be seen from Table 3.

**Table 3:** Confusion matrix and *F*-scores for the sentiment classifier.

Class	Negative (predicted)	Neutral (predicted)	Positive (predicted)
Negative	166	17	6
Neutral	18	197	12
Positive	10	20	134
*F*-score	0.87	0.85	0.85

### Topic Modeling

We performed topic modeling using a total of 3286 preprocessed tweets with a negative sentiment: 1770 (53.9%) tweets came from the manually annotated data set, and another 1516 (46.1%) tweets came as a result of automatic classification. We made this data set available on our GitHub repository [63].

**Figure 6:**
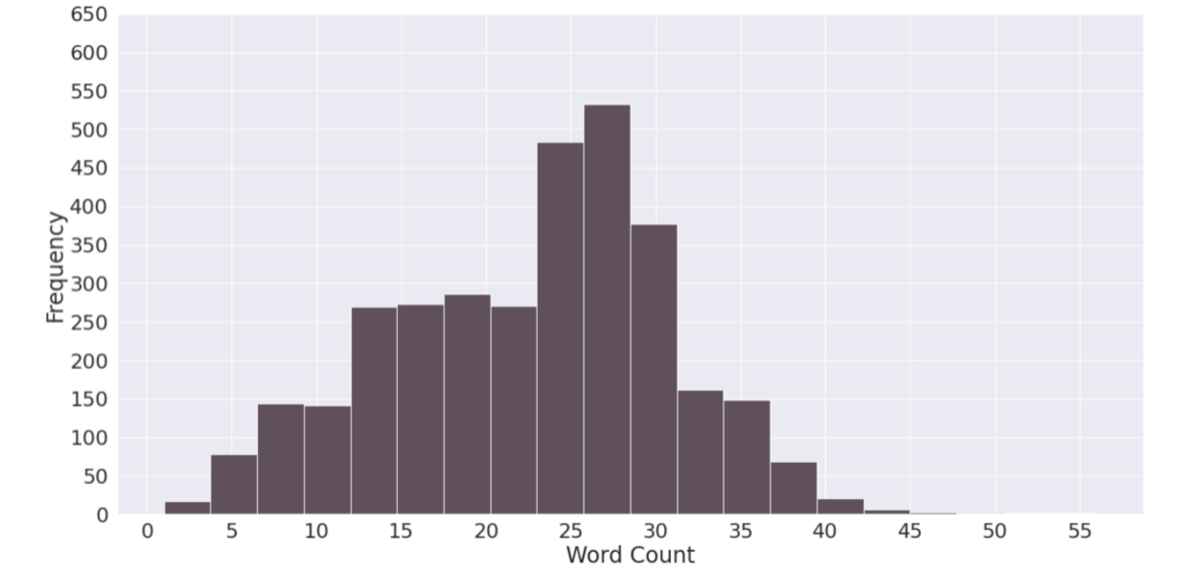
Tweet word count distribution.

The average word count in the data set was 22, with an SD of 8 words. The word count distribution in negative tweets can be seen in Figure 6. The distribution was slightly negatively skewed, but overall, it was a normal distribution, with the 25th percentile at 16 words and the 75th percentile at 28 words.

**Figure 7:**
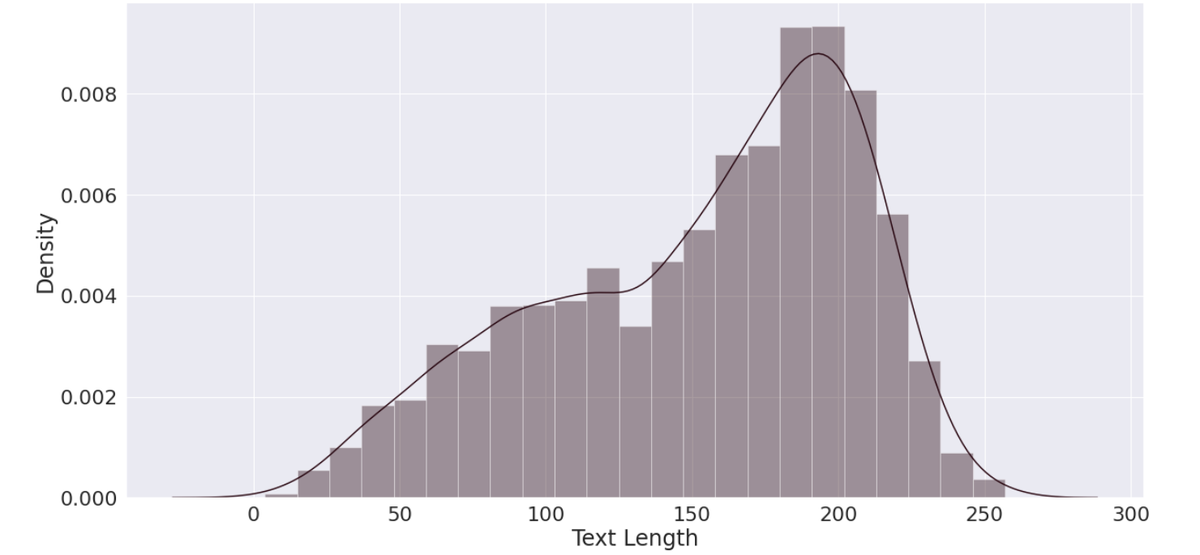
Tweet length distribution.

The text length distribution can be seen in Figure 7. It was also negatively skewed but more significantly than the word count distribution, with an average length of 152 characters and an SD of 53 characters. The length of tweets was often connected with the nature of the negative sentiment, which affected the grouping of such tweets into a certain topic.

**Figure 8:**
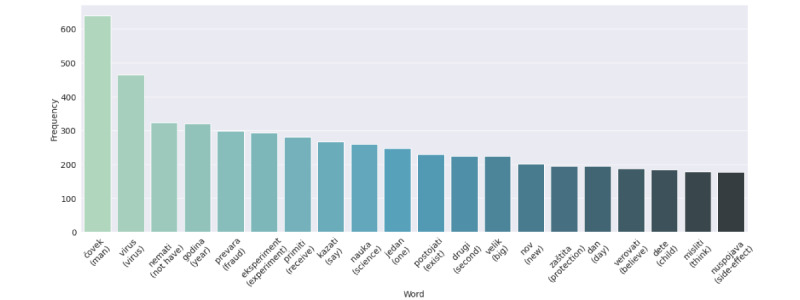
Frequency of top 20 words.

Figure 8 displays the 20 most frequent words in the preprocessed data set. The top 20 words included the terms “virus,” “fraud,” and “experiment,” proving that the most frequent words reflect the nature of the data set consisting of tweets with a negative sentiment regarding vaccination.

#### Topic Analysis

The optimal number of topics that we obtained for LDA and NMF was 14 and 13, respectively.

The direct output of both models were the most prominent keywords for each of the topics. We defined the topic names by first looking at the top 20 keywords per topic and then by checking the name against the 30 most prominent tweets assigned to that topic. The defined names and top 10 words per topic for both models can be seen in the table in Multimedia Appendix 1. To display the topics in the LDA method, we used the *Gensim* method “show topics,” which returns an arbitrary order of topics. For NMF, like in the case of LDA, there is no natural ordering of topics. The topics are inferred from the highest frequency of words per topic using the topic-word H matrix, which can give us an idea of the content of the topic.

Since we were interested in the topics that are most discussed in our data set, hoping that they would also point to the main reasons for vaccination hesitancy, we ranked the obtained topics by importance by extracting the number of tweets in which each topic was dominant. This topic ranking is presented in Table 4, along with the original topic number. We analyzed each of the topics based on the ordering in this table.

Based on the number of tweets in Table 4, we can see that both methods generate 1 dominant topic. In the case of LDA, 692 (21.1%) tweets belonged to topic 1, and in the case of NMF, 606 (18.4%) tweets belonged to topic 13.

The 2 main ideas that appear in the first few dominant topics can be shortly summarized as concern over vaccine effectiveness and side effects. These ideas are often brought together into consideration. The dominant topic for the LDA method contains these topics mixed. NMF succeeded in extracting a dominant topic based on these 2 ideas, with an emphasis on the concern over the vaccination of children. Even though the word “child” appears among the LDA keywords, there were almost no tweets regarding the vaccination of children in the first 30 most important tweets for that topic. These 2 main ideas were later identified by LDA as several separate topics (topics 3, 4, 10, 11, and 13).

The second-most dominant topic for both LDA (n=420, 12.8%) and NMF (n=279, 8.5%) can be described as doubt about the effectiveness of COVID-19 vaccines. There are several subtopics with regard to vaccine effectiveness. NMF results point out the concern about the effectiveness in the context of new COVID-19 strains that appear rapidly due to the massive scale of the outbreak of the pandemic. In topic 3, LDA struggled with several subtopic mixtures. In the first subtopic, we can see the belief that the vaccine is less effective than natural immunity, while the second subtopic is more about side effects. Once again, we notice the failure of LDA to separate these topics. NMF extracted the topic of natural immunity as a separate topic (topic 8).

The third dominant topic for NMF is the negative sentiment toward government politics related to pandemics. This is not strictly an opinion or attitude toward vaccination, but it often happens to seem so due to the attitudes of Twitter users about government policies in general. As already mentioned in the Relevance Classifier section, it was difficult to draw a strict boundary between political opinions and vaccination itself.

Subtopics in this topic may include frustration over the freedom of movement and choice regarding vaccination, the belief that government institutions are not competent enough in the fight against pandemics, and the belief that their decisions are influenced by various global powers. The third dominant LDA topic pointed out users’ frustration with the loss of freedom of movement and freedom of vaccination choice but again got mixed by the skepticism toward medical science, which formed a separate topic found by NMF (topic 5). Therefore, the fourth-most dominant topic found by NMF was skepticism toward vaccine effectiveness connected to the skepticism toward official scientific institutions and experts.

The next, fifth topic given by NMF is skepticism toward the effectiveness of the vaccines in the context of natural immunity. The thesis is that it is better to build immunity naturally than through the vaccination process. This was covered as a subtopic in the second dominant topic given by the LDA method.

The sixth and seventh topics given by the NMF method present a concern that vaccines were fast to appear and therefore could not have been sufficiently tested. This thesis appears in many topics given by the LDA method but was most pronounced in the topic 8.

The eighth dominant topic by NMF presents a pronounced fear of vaccination side effects, including death. Specific side effects are dominant in topic 10 found by LDA. Similarly, the next topic given by the NMF method outlines concerns about so many booster doses, which hints to users either that vaccines are not effective enough or that such a high number of doses may produce heavier side effects, which is the main concern in topic 11 in LDA.

Here, we must outline that the LDA method isolated a topic about the fear regarding messenger RNA (mRNA)–based vaccines (topic 13). The fear is connected with their effectiveness but mainly with the side effects, since in tweets, mRNA vaccines are often connected to genetic treatments. These types of vaccines are often connected with conspiracy theories that some center of power has a genetic mutation agenda for some kind of population control. This was a well-defined topic in both models (LDA topic 12, NMF topic 7).

For the NMF method, 4 last topics exposed fears that the entire pandemic and vaccination process are somehow conspired by various centers of power and for various reasons. The tenth topic postulates that COVID-19 exists only in the media, and topics 11 and 12 postulate that vaccines are a fraud for various different reasons (profit, population control, etc). These concerns appear in topics 6, 9, and 14 in the LDA method.

In the end, NMF extracted a general topic that encompasses frustration with key decision makers in the context of the pandemic. It is a more general version of topics 2, 5, and 6 in LDA.

**Table 4:** LDA^a^ and NMF^b^ topics by number of tweets (N=3286).

LDA				NMF		
Topic number	Topic name	Tweets, n (%)	Topic number		Topic name	Tweets, n (%)
1	General concern over vaccine effectiveness and side effects	692 (21.1)	13		Concern over vaccine side effects: negative attitude toward vaccination of children and anxiety about the effects on their health	606 (18.4)
3	Doubt about effectiveness: natural immunity is a better protection, and side effects overweigh benefits	420 (12.8)	6		Doubt about effectiveness, especially for new strains	279 (8.5)
2	Mistrust of science and concern over violation of freedom of choice and movement	329 (10.0)	12		Linking vaccination with the negative attitude toward the country politics	272 (8.3)
8	Vaccines are an experiment	314 (9.6)	5		Mistrust of science and experts	271 (8.2)
4	Doubt about vaccine effectiveness: vaccines are no protection, especially regarding new strains	264 (8.0)	8		Doubt about vaccine effectiveness: natural immunity is better protection	263 (8.0)
7	Conspiracy theory: COVID-19 is a fraud; vaccines change the DNA	238 (7.2)	4		Vaccine is an experiment and is insufficiently tested	251 (7.6)
6	Vaccines and other measures are means of spreading fear and a money-making scheme	235 (7.2)	9		Anxiety over short vaccine development time and, consequently vaccine side effects	243 (7.4)
12	Conspiracy theory: vaccine as a means of population reduction and control	166 (5.1)	1		Pronounced fear of different vaccine side effects, primarily death	230 (7.0)
5	Mistrust of the government and institutions	146 (4.4)	10		Doubt about vaccine effectiveness and anxiety over side effects due to having to take boosters	218 (6.6)
13	Fear of side effects: vaccines are insufficiently tested, especially the mRNA^c^ technology	119 (3.6)	11		Conspiracy theory: COVID-19 does not exist, and consequently, vaccines are a fraud	209 (6.4)
9	Conspiracy theory: Vaccines are a global fraud	100 (3.0)	2		Conspiracy theory: vaccines are a fraud	199 (6.1)
14	Conspiracy theory: linking vaccines with world powers and their agendas	95 (2.9)	7		Conspiracy theory: vaccine as a means of population reduction and control	134 (4.1)
10	Fear of specific side effects	91 (2.8)	3		General frustration over vaccines, institutions, and power players	111 (3.4)
11	Doubt about effectiveness: questioning the need for boosters	77 (2.3)	N/A^d^		N/A	N/A

^a^LDA: latent Dirichlet allocation.

^b^NMF: nonnegative matrix factorization.

^c^mRNA: messenger RNA.

^d^N/A: not applicable.

## Discussion

### Principal Findings

In this study, we demonstrated the application of several NLP techniques used in combination to find hidden concerns regarding COVID-19 vaccination to a data set of tweets in Serbian. We used BERT-based classifiers to augment the manually annotated data set and obtain the final data set of tweets expressing a negative sentiment toward the COVID-19 vaccination process. We then performed topic modeling on this subset using LDA and NMF and combined the topics obtained by both methods to compile a list of 5 overarching reasons for vaccine hesitancy in Serbia.

### Automatic Tweet Classifier

In addition to being able to correctly classify tweets according to their relevance and sentiment, we also wanted to analyze human annotation errors. For both classifiers, we found that there were cases where human annotators made errors, which was to be expected, given the semantic complexity of the tweets. However, the algorithms proved to be resilient to this syndrome and statistically learned well from the majority of correctly labeled examples. To confirm this conclusion, we carefully revised annotations for the test set to the point where we could claim that the test set was almost fully correctly annotated. Nevertheless, we drew conclusions about the confused examples from the original test set.

Upon closer inspection, it was confirmed that this type of annotation task was difficult for people to perform and to decide objectively and with utmost certainty which labels to assign. As mentioned earlier, the algorithm often outperformed its supervisor by about 12%. This led to the conclusion that annotation was an emotionally and mentally difficult process in which the annotator made typical human mistakes. BERTić, however, learned statistically from the majority of correctly labeled examples. Nevertheless, there was overfitting present in the fine-tuning process, indicated by extremely high training accuracy. This indicates that more data would improve the algorithm. The supervisor outperformed the algorithm in about 8% of the examples. These are the examples that usually contain complex emotional content and figurative language. For many of these examples, broader knowledge is required. Clearly mixed cases accounted for 12%. These examples are mostly long tweets with multiple contradictory statements. Any disagreement is therefore justified. Further inclusion of intermediate values would likely lead to improvement on this basis.

All this suggests that the algorithm would improve if we were to apply some revised annotations through the so-called active learning approach [64]. The already explained overfitting in combination with the annotators’ mistakes may lead to a slight bias and degradation of the overall performance of the classifier. However, we expect this to produce a weak effect since most examples are correctly labeled and the algorithm learns robustly and statistically from most correctly labeled examples.

The most similar classifier in the literature for the English language was reported by To et al [9]. Several classifiers were analyzed and compared in this paper. The BERT-based model was reported to have the highest performance. Our metrics values are slightly lower. This is expected because our classifier is more complex as it categorized tweets into several classes according to relevancy and sentiment, whereas classifiers in Ref. [9] are trained in a binary fashion, dividing tweets into negative sentiments and others. Our approach may serve better future work that may encompass the analysis of positive-sentiment tweets.

### Topic Modeling

Even though LDA is a generative model, in text mining it introduces a way to attach topical content to text documents. It views each document as a mixture of multiple distinct topics. Our tweets do not fulfill this requirement as they are usually short documents with 1 dominant topic. In addition, LDA suffers from order effects, meaning that different topics can be generated when the order of training data is shuffled. This error can lead to misleading results: the words that define the topic or the order of their importance can be different, which leads to a difference in defining the topic name. As a consequence, there is also a change in the distribution of topics in the documents.

NMF is a linear-algebraic model that factors high-dimensional vectors into a low-dimensional representation. Similar to principal component analysis, NMF takes advantage of the fact that the vectors are nonnegative. It works best with shorter texts, such as tweets or titles, because it does not predefine a document as a mixture of different topics but rather describes it through latent features, which are further clustered.

Having these short descriptions of the used models in mind, along with the analysis of the topics given in the previous section, we can conclude that NMF gave us clearer and more defined topics when looking at the output: keywords and most prominent tweets per topic. However, the LDA-specific results should not be omitted when considering the reasons for vaccination hesitancy, especially since they highlight some aspects that are not immediately seen in NMF topics. Therefore, we compiled the following list of reasons the users of Twitter in Serbia could be hesitant about COVID-19 vaccination by summarizing the topics in both models in the order of their importance:

- Concern over vaccine side effects: (1) general side effects, (2) side effects for children, (3) side effects due to many required doses
- Concern over vaccine effectiveness: (1) natural immunity is better protection, (2) vaccines are not effective against new COVID-19 strains, (3) vaccines are not effective since so many doses are required
- Concern over insufficiently tested vaccines: (1) side effects of such vaccines, (2) effectiveness of such vaccines, (3) violation of freedom by imposing the use of such vaccines
- Mistrust of authorities: (1) medical experts and institutions, (2) government and political decision makers
- Conspiracy theories: (1) vaccines are a money-making scheme; (2) vaccines, especially mRNA vaccines, change DNA; (3) COVID-19 does not exist; thus, vaccines are unnecessary; (4) vaccines are a means of population reduction and control; (5) vaccines are an instrument of world powers and their agendas

Both Table 4 and the table in Multimedia Appendix 1 remain insightful for anyone needing a more detailed overview of people’s concerns regarding the vaccination process.

### Conclusion

This paper presents a combination of NLP methods aimed at studying the reasons for vaccine hesitancy in Serbia. It focuses on information collected from Twitter and expressed by Twitter users. We first gathered tweets with keywords regarding COVID-19 vaccination. Some of the gathered tweets were used to build a BERT-based classifier for automatic detection of tweets with a relevant and negative opinion about the immunization process. We then used this classifier to automatically classify the second part of the tweets. The technology we used to build this classifier, based on the transformer encoder architecture BERTić, showed prominent and high-quality results. The classifier we built can be used effectively in future studies of public opinion and in particular the immunization process as the world is still unsure about the way pandemics will evolve. Our approach can be relatively easily extended to other world languages.

The second part of the analysis consisted of applying topic modeling methods, LDA and NMF, to negative-sentiment tweets. We considered using the resulting BERTić architecture to perform topic analysis. However, embeddings obtained in such a way did not behave as expected during clustering. In future work, we plan to consider the obtained sentiment classifier for the task of topic modeling. Specifically, our plan is to use sentence-BERT [65] to obtain tweet embeddings and further cluster them into topics. Given that such resources have not yet been built for South Slavic languages, we opted for using the combination of more traditional techniques for topic analysis.

We isolated and listed the dominant topics in the tweets with a negative sentiment toward vaccination. The main result of this paper is seen in well-researched reasons behind the negative sentiments toward vaccination. Given these reasons, it is now possible to better understand the concerns of people regarding the vaccination process. This will allow the government and medical and pharmaceutical institutions to develop or redefine educational strategies that better address these issues. We hope this can significantly increase the effectiveness of the fight against the COVID-19 pandemic.

## Appendix

Multimedia Appendix 1 Topics and top 10 keywords detected by latent Dirichlet allocation (LDA) and nonnegative matrix factorization (NMF).

## Abbreviations

API: application programming interface
BERT: bidirectional encoder representations from transformers
LDA: latent Dirichlet allocation
mRNA: messenger RNA
NLP: natural language processing
NMF: nonnegative matrix factorization
RNN: recurrent neural network
TF-IDF: term frequency–inverse document frequency
WHO: World Health Organization

## Data Availability

The data set we used to perform topic modeling is available on our GitHub repository [63].
