# Supplementary material for: Uncovering the Reasons Behind COVID-19 Vaccine Hesitancy in Serbia: Sentiment-Based Topic Modeling
Source: J Med Internet Res. 2022 Nov 17;24(11):e42261. doi: 10.2196/42261 (PMC9671489; doi:10.2196/42261)
Supplement: Multimedia Appendix 1 [file jmir_v24i11e42261_app1.docx]

Table 1 Topics and top 10 keywords detected by LDA and NMF.

|  | **LDA topics** | **Top 10 words** | **NMF topics** | **Top 10 words** |
| --- | --- | --- | --- | --- |
| 1 | **General concern over vaccine effectiveness and side-effects** | godina (year), čovek (man), primiti (receive), dan (day), dete (child), jedan (one), drugi (second), umreti (die), trebati (need), kazati (say) | **Pronounced fear of different vaccine side-effects, primarily death** | čovek (man), umirati (die), umreti (die), problem (problem), misliti (think), trebati (need), smrt (death), svet (world), posledica (consequence), videti (see) |
| 2 | **Mistrust of science and concern over violation of freedom of choice and movement** | nauka (science), - (expletive), nemati (not have), čovek (man), zdravlje (health), kazati (say), svet (world), doktor (doctor), želeti (want), dovoljno (enough) | **Conspiracy theory: Vaccines are a fraud** | prevara (fraud), velik (big), istorija (history), laž (lie), jasno (clearly), tv (TV), svinjski (swine), videti (see), verovati (believe), dokazati (prove) |
| 3 | **Doubt about effectiveness: Natural immunity is a better protection and side-effects overweigh benefits** | nemati (not have), virus (virus), zaštita (protection), čovek (man), bolest (illness), drugi (second), simptom (symptom), nikakav (of no sort), štititi (protect), misliti (think) | **General frustration over vaccines, institutions and power players** | - (expletive), dati (give), - (expletive), - (expletive), mozak (brain), voda (water), piti (to drink), otići (to leave), maska (mask), dobro (well) |
| 4 | **Doubt about vaccine effectiveness: Vaccines are no protection, especially regarding new strains** | nov (new), virus (virus), soj (strain), reći (say), srbija (Serbia), omikron (Omicron), zaraziti (infect), mutacija (mutation), zaraza (infection), broj (number) | **Vaccine is an experiment and is insufficiently tested** | eksperiment (experiment), medicinski (medical), genski (genetic), eksperimentalan (experimental), ispitivanje (examination), faza (phase), kineski (Chinese), vršiti (perform), pfizer (Pfizer), odobriti (approve) |
| 5 | **Mistrust of the government and institutions** | priča (story), sns (SNS), virus (virus), smrtnost (mortality), smrt (death), bakterija (bacteria), država (country), podatak (information), zdravstven (health), slučaj (case) | **Mistrust of science and experts** | nauka (science), kazati (say), verovati (believe), nemati (not have), doktor (doctor), medicina (medicine), dokazati (prove), misliti (think), pitanje (question), struka (experts) |
| 6 | **Vaccines and other measures are means of spreading fear and a money-making scheme** | postojati (exist), čovek (man), daleko (far), posledica (consequence), eksperiment (experiment), problem (problem), služiti (serve), videti (see), mera (measure), obavezan (mandatory) | **Doubt about effectiveness, especially for new strains** | virus (virus), nov (new), soj (strain), napraviti (make), omikron (Omicron), reći (say), mutacija (mutation), mutirati (mutate), grip (flu), hiv (HIV) |
| 7 | **Conspiracy theory: COVID-19 is a fraud, vaccines change the DNA** | prevara (fraud), verovati (believe), dnk (DNA), virus (virus), otrov (poison), postojati (exist), velik (big), menjati (change), pitanje (question), nauka (science) | **Conspiracy theory: Vaccine as a means of population reduction and control** | gejts (Gates), bil (Bill), čip (chip), populacija (population), čipovati (to chip), smanjiti (to decrease), čovečanstvo (humankind), zemlja (country), kazati (say), - (expletive) |
| 8 | **Vaccines are an experiment** | eksperiment (experiment), ispitivanje (examination), eksperimentalan (experimental), nuspojava (side-effect), kineski (Chinese), primiti (receive), morati (must), faza (phase), medicinski (medical), proizvođač (producer) | **Doubt about vaccine effectiveness: Natural immunity is a better protection** | zaštita (protection), imunitet (immunity), štititi (protect), dobar (good), nikakav (of no sort), maska (mask), nemati (not have), značiti (to mean), prirodan (natural), misliti (think) |
| 9 | **Conspiracy theory: Vaccines are a global fraud** | milijarda (billion), imun (immune), sistem (system), otrovan (poisonous), globus (globe), fašist (fascist), sns (SNS), srbija (Serbia), ozbiljan (serious), rnk (RNA) | **Anxiety over short vaccine development time, and consequently vaccine side-effects** | godina (year), dan (day), dva (two), umreti (die), prošli (past), mesec (month), dobiti (get), proći (to pass), prvi (first), raditi (to work) |
| 10 | **Fear of specific side-effects** | srčan (heart), izazivati (to cause), udar ((heart) attact), imunitet (immunity), studija (study), rizik (risk), sportist (athlete), upozoravati (warn), masovan (mass), tromb ((blood) clot) | **Doubt about vaccine effectiveness and anxiety over side-effects due to having to take boosters** | primiti (receive), doza (dose), treći (third), verovati (believe), dobiti (get), morati (must), prvi (first), trebati (need), smetati (to bother), tri (three) |
| 11 | **Doubt about effectiveness: Questioning the need for boosters** | doza (dose), test (test), treći (third), primiti (receive), pcr (PCR), pozitivan (positive), drugi (second), propusnica (pass), avion (plane), priznati (admit) | **Conspiracy theory: COVID-19 virus does not exist and consequently, vaccines are a fraud** | postojati (exist), antivakser (anti-vaxxer), lek (medicine), otrov (poison), maska (mask), ubiti (kill), laž (lie), ubijati (kill), lažan (fake), mera (measure) |
| 12 | **Conspiracy theory: Vaccine as a means of population reduction and control** | gejts (Gates), laž (lie), bil (Bill), cilj (aim), populacija (population), čip (chip), sredstvo (means), čovek (man), pandemija (pandemic), zemlja (country) | **Linking vaccination with the negative attitude towards the country politics** | sns (SNS), dnk (DNA), menjati (change), srbija (Serbia), kazati (say), rnk (RNA), videti (see), kolonija (colony), član (member), pfizer (Pfizer) |
| 13 | **Fear of side-effects: Vaccines are insufficiently tested, especially the mRNA technology** | dr (dr.), mrn (mRNA), štab (response team), terapija (therapy), genski (genetic), krizan (crisis), lek (medicine), lekar (doctor), rak (cancer), kazati (say) | **Negative attitude towards vaccination of children and anxiety about the effects on their health** | jedan (one), dete (child), drugi (second), velik (big), nuspojava (side-effect), bolest (illness), isti (same), lekar (doktor), šteta (damage), mrn (mRNA) |
| 14 | **Conspiracy theory: Linking vaccines with world powers and their agendas** | izbor (choice), trebati (need), pasoš (passport), - (expletive), živeti (live), teror (terror), novak (Novak), - (expletive), srbin (Serbian), medicinski (medical) | N/A | N/A |
